# Cooperative and Individual Mandala Drawing Have Different Effects on Mindfulness, Spirituality, and Subjective Well-Being

**DOI:** 10.3389/fpsyg.2020.564430

**Published:** 2020-10-09

**Authors:** Chao Liu, Hao Chen, Chia-Yi Liu, Rung-Tai Lin, Wen-Ko Chiou

**Affiliations:** ^1^College of Aviation, Hua Qiao University, Xiamen, China; ^2^Graduate Institute of Business and Management, Chang Gung University, Taoyuan, Taiwan; ^3^Department of Psychiatry, Chang Gung Memorial Hospital, Taipei, Taiwan; ^4^Graduate School of Creative Industry Design, National Taiwan University of Arts, New Taipei City, Taiwan; ^5^Department of Industrial Design, Chang Gung University, Taoyuan, Taiwan

**Keywords:** positive psychology, mandala drawings, mindfulness, spirituality, subjective well-being

## Abstract

Mandala drawing was first practiced by Tibetan buddhists and then developed by Carl Gustav Jung, who felt certain that mandala drawing has the function of integrating psychological division, enhancing psychological harmony, and preserving personality integrity. Previous studies on mandala drawing have mainly focused on alleviating people’s negative emotions, such as anxiety and depression. Therefore, this study explored the effect and mechanism of mandala drawing on the improvement of subjective well-being (SWB), mindfulness, and spirituality from positive psychology’s viewpoint and compared the different effects of cooperative mandala drawing (CMD) and individual mandala drawing (IMD) on mindfulness, spirituality, and SWB. A total of 76 students were recruited from Chang Gung University, and the aforementioned three main variables were measured before and after the coloring experiment. The results indicated that both CMD and IMD significantly enhanced the subjects’ spirituality. Compared with IMD, CMD has a more significant improvement and promotion effect on SWB of subjects by affecting PA, while IMD had no significant effect on PA, and the enhancement effect of SWB was weaker than that of CMD. Mindfulness, spirituality, and SWB all positively correlated with each other. This study highlights the mechanism of mandala drawing and the theoretical understanding of the relationship between mindfulness and SWB. Mandala drawing especially CMD has a positive effect on spirituality and SWB, which may provide individuals with a simple and easy method to improve their happiness.

## Introduction

Positive psychology (PP) was founded by American psychologists Seligman and Csikzentmihaly in 2000. Positive psychological interventions (PPI) are psychotherapy guided by positive psychology. It is defined as consciously increasing the cognition or behavior of positive state (such as positive emotion and life satisfaction), rather than reducing negative state (such as depression and anxiety). In short, PPI is a kind of psychological intervention to promote positive outcomes through positive processes (Seligman and Csikszentmihalyi, 2014). PPI aims to promote happiness and indirectly reduce the severity of mental pain. PPI is not simply to tell people to be positive or happy. Instead, it is a specific strategy that allows people to often improve their happiness through indirect means, such as finding their own strengths or engaging in acts of kindness (Snyder and Lopez, 2001).

With the development of positive psychology, Wong then proposed positive psychology 2.0 (PP2.0). First of all, PP2.0 pointed out that life is full of both suffering and happiness, and it is impossible to get real happiness if only valuing the guidance of happiness or ignoring the search for the cause of suffering. Not looking at the suffering is a paradox that is both inhuman and emotionally impossible (Wong, 2011). When there is suffering, it does not necessarily mean that there is no happiness, but to transform all kinds of suffering with meaning and mindfulness (Wong, 2019). Secondly, PP2.0 believes that every positive emotion or experience has its disadvantages, while every negative emotion or experience has its advantages. PP2.0 provides a dialectical, balanced, and cross-cultural perspective. More recently, Wong’s (2020) dual system model has illuminated the mechanisms for achieving a better life, not by emphasizing positive and avoiding negative attitudes but by embracing and integrating positive and negative experiences. Finally, PP2.0 believes that positive psychology should incorporate indigenous psychology (Wong, 2016).

According to the source of the stimulus, the sensation can be divided into external sensation and interoception. External sensation is the sensation caused by external stimuli acting on the sensory organs, including vision, hearing, anger, taste, and skin sensation. Interoceptions are the sensations caused by internal stimulus in an organism, including motor sensations, balance sensations, and visceral sensations such as hunger, thirst, fullness, and suffocation. Interoception is an essential feature of the human nervous system and is associated with many biological and psychological phenomena such as eating, craving, and decision-making (Barrett and Simmons, 2015). PP2.0 does not rely entirely on external stimulation to treat emotions. Besides external feelings, there are also interoceptions. Liu previously found that loving kindness meditation could improve the interoception of flight attendants and reduce the negative emotions generated by their emotional labor (Liu et al., 2020). In addition to loving kindness meditation, there are also mandala drawings in Oriental indigenous psychology. Mandala drawing is a form of comprehensive physical and mental training. Mandala drawing, first practiced by Tibetan buddhists and then developed by Carl Jung, is a form of integrative body–mind training and currently one of the main forms of drawing psychotherapy. Jung believed that mandala drawing has the function of integrating psychological division, enhancing psychological harmony, and preserving personality integrity (Jung, 2012). Advocated by Jung (2012), mandala drawing has become one of the most important and widely used forms of artistic expression therapy. Previous researches on the use of mandala drawing for psychotherapy have demonstrated their effectiveness in different populations.

First of all, research on the application of art therapy for mandala drawing in the general population has found that it can identify psychological disorders (Kim et al., 2014), reduce depression and anxiety in female college students (Flett et al., 2017), and reduce anxiety in college students (Hass-Cohen et al., 2018). Second, the application of mandala drawing art therapy to people with physical and mental disabilities has been shown to reduce anxiety in people with intellectual disabilities (Schrade et al., 2011) and patients with breast cancer (Elkis-Abuhoff et al., 2009). However, in addition to studying the use of mandala drawing to alleviate negative emotions such as anxiety and depression, it is essential to study whether mandala drawing can raise happiness and to comprehend the mechanism of happiness from positive psychology’s viewpoint. Mindfulness and spirituality are both correlated with subjective well-being (SWB) (Csikszentmihalyi, 2013). However, only several studies have examined whether mandala drawing art therapy can increase mindfulness, spirituality, and SWB. The little literature that exists currently does indicate that mandala drawing can increase young people’s mindfulness (Carsley and Heath, 2018), increase the spirituality of nursing staff (Yilmaz et al., 2018), and have a positive effect on SWB (Shiah and Hwang, 2019). Therefore, the objective of this study is to verify whether mandala drawing can increase mindfulness, spirituality, and SWB from a positive perspective.

Traditional meditation and Mandala drawing are both individual behavioral activities, which are the research scope of positive psychology 1.0 (PP1.0). PP 1.0 excludes the causes and functions of negative experience, relies on the internal concentration of social reality, fails to integrate negative experience, and does not encourage the understanding of real conflict interaction. It is based on avoiding or reducing attention to social interaction (Wong, 2011). Traditional mandala drawing has the risk of disconnection from reality and hinders people’s understanding of social reality. It has many similarities with avoidance mechanism, which aims at limited interaction with reality and away from interaction with wider social conflicts (Chiou et al., 2020). In the perspective of PP2.0, Liang devised a new design of Jung’s individual mandala drawing (IMD), dividing a full circle into various sections, to create an intervention called the cooperative mandala drawing (CMD). The practice of CMD, through the collaborative creation of two or more people, allows teenagers not only to express their inner selves but also to present the spiritual reality of creating a mandala process with others, helping them to generate positive psychology, and also to symphony with the group, and to experience the beauty of unity (Liang et al., 2020). When doing CMD, one can feel the way others think, the mood, and then add their own ideas. Cooperating with others, it is not lonely and has a richer sense of surprise. On the one hand, collaborative mandala drawing brings the satisfaction of personal mandala drawing, and on the other hand, it brings the thoughtfulness that you want to give when interacting with others. Collaborative mandala drawing cannot only express yourself but also help each other, imitate, create, and learn autonomously. In the process, it can enhance the motivation of team collaboration and the fun and development of altruism interpersonal relationship (Chen H. et al., 2019). In Liang’s study, it was observed that the subjects showed a more focused state of engagement and absorption, and fuller positive emotions during CMD activities than IMD, and from this it was suggested that CMD may help individuals to enter higher levels state of mindfulness and a more pleasant experience for individuals.

SWB mainly refers to people’s overall emotional and cognitive evaluation of their quality of life. In this sense, what determines whether people are happy is not what actually happens—the key is how people interpret what is happening emotionally and how they cognitively process this emotion (Diener et al., 2002). SWB consists of two parts: emotional equilibrium and life satisfaction. Emotional equilibrium refers to an overall and general valuation of one’s life, where happy experiences occupy a comparative advantage in the person’s mind compared with unpleasant emotional experiences. Emotional equilibrium contains both positive emotions and negative emotions. However, these two attributes are not necessarily correlated, and they are two comparatively independent variables. Life satisfaction is an individual’s comprehensive judgment of life (Diener et al., 1999). SWB also has a negative effect on depression and anxiety. Therefore, the higher SWB is, the lower the severity of depression or anxiety of an individual is (Malone and Wachholtz, 2018). Due to the importance of SWB, we should study how to enhance its emotional processes and cognitive processes, which are correlated with spirituality and mindfulness (Csikszentmihalyi, 2013).

Mindfulness is the purposeful and conscious awareness of what is happening at the present without any judgment, analysis, or reaction (Kabat-Zinn, 2003). Researches have shown that mindfulness can improve the SWB of aged people (Aliche and Onyishi, 2019), workplace employees (Allen et al., 2017), and teachers (Hwang et al., 2017). Several researchers have also confirmed that college students with a higher level of mindfulness have a higher level of SWB (Bajaj and Pande, 2016; Ge et al., 2019). Although there exists considerable evidence indicating that mindfulness has a positive effect on SWB, there is limited research to explore the mechanisms behind this relationship. Another underexplored relationship is how spirituality may be linked to further explanations of mindfulness and SWB.

Spirituality refers to people’s pursuit and experience of the connection with the essence of life. It has three dimensions: a connection to oneself, a connection to others and nature, and a connection to transcendence (Meezenbroek et al., 2012). Studies have revealed that spirituality can improve SWB (Kamitsis and Francis, 2013), particularly in elderly people (Lifshitz et al., 2019) and teenagers (Sarriera et al., 2014). Another research explored spiritual prepositional variables and showed how mindfulness can improve individual spiritual growth (Matiz et al., 2018). Researchers have examined specific groups of people and determined that mindfulness can improve spirituality in teenagers (Cobb et al., 2015), improve the spirituality of patients with late-stage cancer (Casula, 2018), improve the spirituality of married couples (Lord, 2017), and have a positive effect on the spirituality of patients with psychosis (Da Silva and Pereira, 2017). The literature indicates that there exist reasons to believe that people with a high degree of mindfulness may be able to experience high spirituality and thus increase their SWB.

There is no previous study on the comparative analysis of CMD and IMD; this study is the first one that tested and compared them. Previous studies on CMD are mostly qualitative analysis and exploratory research; this study is also the first one that use data to perform quantitative confirmatory analysis on CMD. Thus, the research purpose of this study is to explore the different impacts of CMD and IMD on mindfulness, spirituality, and SWB from the viewpoint of PP 2.0.

According to the aforementioned theories, we established the following hypotheses:

Hypothesis 1a. In both cooperative group and individual group of Mandala drawing, the post-assessed scores of the three questionnaires (spirituality, mindfulness, and SWB) are higher than the pre-assessed scores significantly.
Hypothesis 1b. The post-assessed scores of three questionnaires (spirituality, mindfulness, and SWB) in cooperative group are higher than those in individual groups significantly.

Hypothesis 2. There exists a positive correlation among mindfulness, spirituality, and SWB.

## Materials and Methods

### Participants

The study initially enrolled 80 students from Chang Gung University in Taiwan, and eventually 76 of them met the screening criteria (19 males, 57 females). All participants were aged from 18 to 41 years (Mean = 22.3, *SD* = 4.73). Participants were randomly divided into two groups: the CMD group (36 participants) and the IMD group (40 participants). There was no significant difference in demographic characteristics between the two groups (Table 1).

**TABLE 1:** Demographic characteristics of participants.

**Characteristic**	**Total**	**IMD group**	**CMD group**
Age (SD)	22.30 (4.73)	21.55 (4.43)	23.87 (5.21)
Male (%)	19 (25%)	10 (25%)	9 (25%)
Female (%)	57 (75%)	30 (75%)	27 (75%)

*No demographic characteristic was significantly different among the two groups.*

### Instruments

#### State Mindfulness Scale

This scale was used to measure the participants’ state of mindfulness. The State Mindfulness Scale (SMS) was developed by Tanay and Bernstein (2013) and contains two subscales: mindfulness that reflects the state of the body’s feelings and mindfulness related to psychological events. The body mindfulness subscale has 15 items, and the psychological mindfulness subscale has six items. Thus, the total number of items is 21. The participants’ answers were given on a five-point Likert scale, where 1 means “not at all” and 5 means “very well.” The total score for each subscale is the subscale score, and the addition of the two subscale scores provides the total score. Previous studies have verified that the SMS has good validity (Botrel and Kubler, 2019). Cronbach’s alpha in this study for this scale was 0.95.

#### Spirituality Scale

This scale was used to measure the spirituality of the participants (de Jager Meezenbroek et al., 2012). This scale contains seven subscales: meaning, trust, acceptance, caring for others, connection with nature, transcendence, and spiritual activity (26 items in total). The participants provided answers on a six-point Likert scale, where 1 means “not at all” and 6 means “very well.” Each subscale’s total score is the subscale score, and the addition of the seven subscale scores provides the total score. Previous studies have verified that the Spirituality Scale has good validity (Jirasek and Hurych, 2018). Cronbach’s alpha in this study for this scale was 0.96.

#### Positive and Negative Affect Scale and Satisfaction With Life Scale

SWB consists of two parts, emotional balance and life satisfaction (Diener et al., 2002). Therefore, two scales were employed to measure this concept. First, the Positive and Negative Affect Scale (PANAS) developed by Watson et al. (1988) was used to measure the participants’ positive and negative emotional experiences. This scale contains two subscales: positive emotional experience (10 items) and negative emotional experience (10 items). The participants’ provided answers on a 5-point Likert scale, where 1 means “none at all” and 5 means “all the time.” Each subscale’s total score is the subscale score, and the addition of the two subscales provides the total score. Previous studies have verified that this scale has good validity (Chen Q.S. et al., 2019). Cronbach’s alpha in this study for the PANAS was 0.97. Second, the Satisfaction With Life Scale (SWLS) was used to measure the life satisfaction of the participants (Diener et al., 1985). This scale comprises seven items, and the participants provided answers on a 7-point Likert scale, where 1 means “strongly disagree” and 7 means “strongly agree.” Previous studies have verified that this scale has good validity (Gigantesco et al., 2019). Cronbach’s alpha in this study for the SWLS was 0.95.

### Drawing Materials

Each participant received an A4-sized paper with a blank circular outline and a box of 12 colored pencils. The pencils (German Faber-Castell) came in the following colors: red, pink, rose, orange, yellow, light green, dark green, purple, blue, brown, black, and white.

### Mandala Drawing Intervention

The mandala drawing intervention involved 5 weekly 90-min sessions, which is a group training. Each meeting involves three consecutive stages: (1) a maximum of 20 min of psychological education, such as an introduction to mandala drawing. (2) For 30 min of mandala drawing practice, participants create mandala by answering the question “How am I now” using any painting material of their choice. If the student struggles with the art production process, the art therapist will provide guidance to reduce frustration in the creation process. At the beginning of mandala drawing, it is suggested to use a color pen from the outside to the inside, which will take about 30 min. Most of the participants will be immersed in the painting without exceeding the concentration beyond the line, and the instructions will be reduced gradually. (3) The final discussion stage lasts for 40 min, during which participants can share their experience of drawing mandala with other participants and lecturers. For the IMD intervention, the subjects were instructed to (1) color the blank circular outline and express thoughts and feelings related to it and (2) think and express the images in their mind. For the CMD intervention, the subjects were instructed (1) every four members to create a mandala together in a blank circular outline; (2) to improve the understanding of oneself and others through non-verbal and verbal expression; and (3) to experience how individual efforts contribute to group work through collaborative work.

### Procedure and Design

**FIGURE 1:**
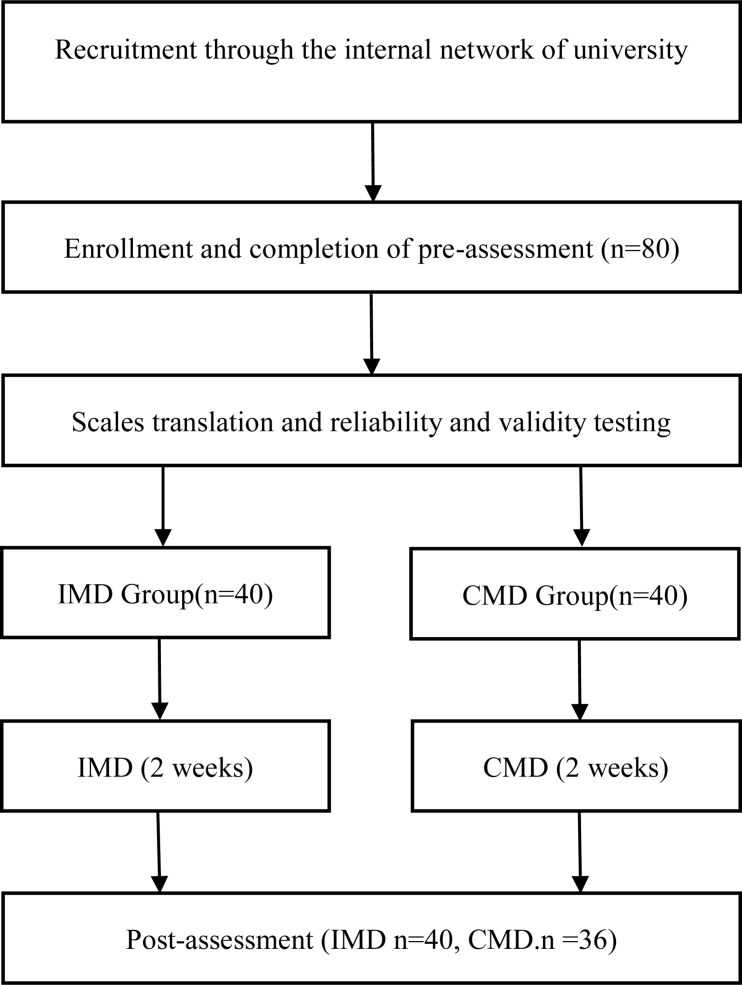
Procedure flowchart. The mean value, standard deviation, and Pearson correlation matrix were used to analyze the data at the description level, and paired-sample *t-*tests was used to analyze the data at the inferential level. The sample size recruited matched or exceeded that of previous studies. Data were analyzed using SPSS version 22 (IBM, 2013), and the significance threshold was set to *p* < 0.05.

We posted a recruitment advertisement for Mandala drawing on the internal network of Changgung University, which shows that this is a self-psychological exploration activity to help college students better understand themselves and solve current problems. Professionals will help participants analyze their Mandala drawings. Students who are interested and qualified to participate in our Mandala drawing research provide their registration information. The criteria for inclusion were (1) adults over 18 years of age and (2) being able to speak and read enough Chinese to complete the questions. The criteria for exclusion were (1) self-reported depression, anxiety, bipolar disorder, suicide, or substance abuse by medical professionals and (2) having had the experience of mandala drawing before. Then, 80 eligible participants were randomly assigned to the experimental group (i.e., the CMD group, 40 participants) or the control group (i.e., the IMD group, 40 participants). This research was carried out by researchers with 12 years of mandala painting practical experience and 3 years of mandala painting teaching experience. The meeting (cooperative or individual mandala painting) was held in a quiet, non-intrusive room, and each subject was given NT $250 at the start of the survey to boost their motivation. At the beginning of the survey, the participants were provided with instructions to make sure they understood them before continuing. After making sure that they understood the instructions, the participants provided demographic information and filled out the following scale: SMS, the Spiritual Attitude and Involvement List (SAIL), PANAS, and SWLS. The time required to answer the scales was about 20 min. The subjects then began a 30-min mandala drawing activity. The duration of the mandala painting intervention was 2 weeks. At the end of the intervention, participants filled out the same questionnaire again and were given an additional NT $250 for participating in this study. In the post-test, two participants were lost in the cooperative group. Following the funnel debriefing procedure, participants were asked if they knew the purpose of the study, what results were investigated, and if they were aware of the same questions in the pre-test and post-test. The funnel debriefing procedure resulted in the exclusion of two participants in the post-test where they expressed a full understanding of the experimental procedure and research purpose, scales of the experiment. In order to enroll subjects who were blind to the experimental conditions and naively drew the mandala, funnel debriefing helped to obtain a homogeneous sample in both groups. Participants have the opportunity to record any number of questionnaires and drawings assigned to them, as well as the anonymity that allows them to withdraw at a later stage and retain their participation. The study was approved by the Chang Gung University Ethics Committee (IRB No: 201901236B0) and carefully reviewed to strictly abide by the ethical guidelines set by the Taiwan Psychological Association (see Figure 1).

## Results

**FIGURE 2:**
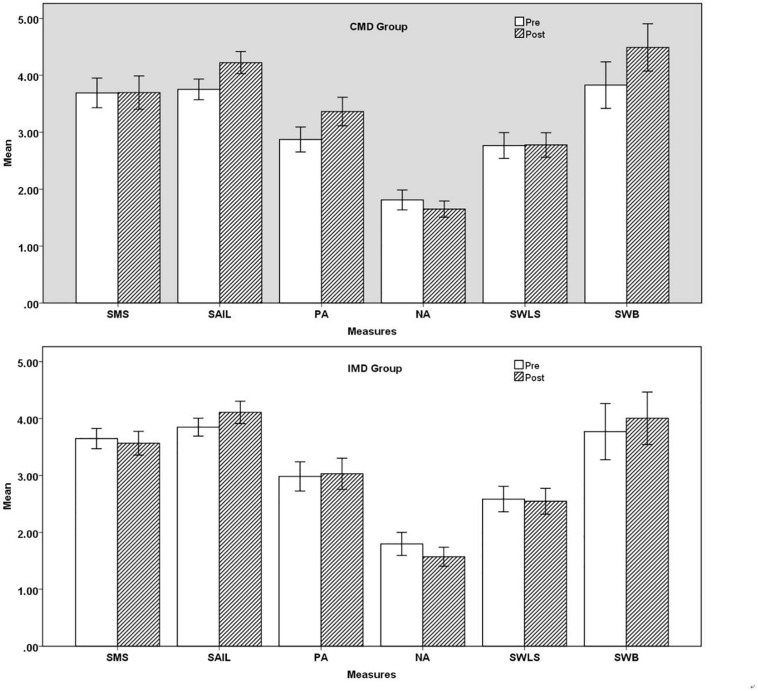
Comparison of six scales between IMD and CMD. SMS, State Mindfulness Scale; SAIL, Spiritual Attitude and Involvement List; SWB, subjective well-being; PA, positive affect; NA, negative affect; SWLS, Satisfaction With Life Scale.

Six 2 (Group Type: CMD, IMD) × 2 (Time: Pre, Post) ANOVA with repeated measures on the Time was conducted on the mindfulness (SMS), spirituality (SAIL), SWB, and the subscales of SWB (PA, NA, and SWLS), in order to explore which part of SWB is influenced by mandala drawing. The P values of Box’s Test results of the three groups of data were all greater than 0.05, indicating that the observed covariance matrices of the dependent variables are equal across groups. The details of the results are presented in Table 2 and Figure 2.

**TABLE 2:** Means and standard deviations for each measure of each group, pre- and post-assessment.

		**Mean (*SD*)**		
**Group**	**Measures**	**Pre**	**Post**	**Post–Pre**
CMD	SMS	3.691 (0.770)	3.696 (0.864)	0.005 (0.510)
	SAIL	3.752 (0.536)	4.222 (0.574)	0.470 (0.229)***
	PA	2.872 (0.650)	3.364 (0.742)	0.492 (0.583)***
	NA	1.811 (0.518)	1.650 (0.421)	−0.161 (0.473)*
	SWLS	2.767 (0.672)	2.776 (0.639)	0.009 (0.100)
	SWB	3.828 (1.208)	4.490 (1.229)	0.662 (0.762)***
IMD	SMS	3.648 (0.553)	3.567 (0.654)	−0.081 (0.525)
	SAIL	3.848 (0.491)	4.108 (0.614)	0.260 (0.432)***
	PA	2.983 (0.804)	3.030 (0.855)	0.047 (0.489)
	NA	1.798 (0.634)	1.573 (0.552)	−0.225 (0.510)**
	SWLS	2.585 (0.701)	2.547 (0.711)	−0.038 (0.226)
	SWB	3.770 (1.542)	4.005 (1.444)	0.235 (0.888)

SMS, State Mindfulness Scale; SAIL, Spiritual Attitude and Involvement List; SWB, subjective well-being; PA, positive affect; NA, negative affect; SWLS, Satisfaction With Life Scale. *p < 0.05; **p < 0.01; ***p < 0.001.

For mindfulness, there was no significant main effect of Time: *F*(1, 74) = 0.411, *p* = 0.523, there was no significant main effect of Group Type: *F*(1, 74) = 0.320, *p* = 0.573, and there was also no significant interaction between Time and Group Type, *F*(1, 74) = 0.526, *p* = 0.471. These results showed that there was no significant difference in SMS scores between the pre-test and the post-test, no significant difference between the CMC group and the FD group, and no interaction effect in the Time × Group Type.

For spirituality, there was a significant main effect of Time: *F*(1, 74) = 82.117, *p* < 0.001 η^2^*_*p*_* = 0.526, and there was no significant main effect of Group Type, *F*(1, 74) = 0.006, *p* = 0.940. However, a significant interaction was found between Time and Group Type, *F*(1, 74) = 6.808, *p* = 0.011, η^2^*_*p*_* = 0.084. Results indicated that both cooperative mandala coloring and free drawing produced a significant increase in SAIL score levels, but cooperative mandala is more effective.

For PA, there was a significant main effect of Time: *F*(1, 74) = 19.201, *p* < 0.001 η^2^*_*p*_* = 0.206, and there was no significant main effect of Group Type, *F*(1, 74) = 0.455, *p* = 0.502. However, a significant interaction was found between Time and Group Type, *F*(1, 74) = 13.031, *p* = 0.001, η^2^*_*p*_* = 0.150. Results indicated that cooperative mandala coloring produced a significant increase in PA score levels.

For NA, there was a significant main effect of Time: *F*(1, 74) = 11.625, *p* = 0.001 η^2^*_*p*_* = 0.136, there was no significant main effect of Group Type, *F*(1, 74) = 0.177, *p* = 0.675, and there was also no significant interaction between Time and Group Type, *F*(1, 74) = 0.318, *p* = 0.574. Results indicated that both cooperative mandala coloring and free drawing produced a significant decrease in NA score levels.

For SWLS, there was no significant main effect of Time: *F*(1, 74) = 0.483, *p* = 0.489, there was no significant main effect of Group Type: *F*(1, 74) = 1.740, *p* = 0.191, and there was also no significant interaction between Time and Group Type, *F*(1, 74) = 1.319, *p* = 0.255. These results showed that there was no significant difference in SWLS scores between the pre-test and the post-test, no significant difference between the CMC group and the FD group, and no interaction effect in the Time × Group Typ.

For SWB, there was a significant main effect of Time: *F*(1, 74) = 22.093, *p* < 0.001 η^2^*_*p*_* = 0.230, and there was no significant main effect of Group Type, *F*(1, 74) = 0.818 *p* = 0.369. However, a significant interaction was found between Time and Group Type, *F*(1, 74) = 5.013, *p* = 0.028, η^2^*_*p*_* = 0.063. Results indicated that cooperative mandala coloring produced a significant increase in SWB score levels.

Hypothesis 2 states that there exists a positive correlation between mindfulness, spirituality, and SWB. The results of the Pearson correlation analysis are presented in Table 3. Specifically, the results revealed a significant negative correlation between negative emotions and SWB (*r* = –0.37, *df* = 78, *p* < 0.05) and no significant correlation between negative emotions and mindfulness, spirituality, positive emotions, or life satisfaction.

**TABLE 3:** Pearson correlation matrix for mindfulness, spirituality, and SWB (post-assessment of mandala drawing).

	**1**	**2**	**3**	**4**	**5**	**6**
1	1					
2	0.66**	1				
3	0.31*	0.51**	1			
4	0.38**	0.54**	0.84**	1		
5	–0.08	0.02	−0.37**	–0.08	1	
6	0.11	0.39**	0.76**	0.45**	0.01	1

1, SMS; 2, SAIL; 3, SWB; 4, PA; 5, NA; 6, SWLS. *p < 0.05; **p < 0.01.

There existed a significant positive correlation between positive emotions and mindfulness (*r* = 0.38, *df* = 78, *p* < 0.05), positive emotions and spirituality (*r* = 0.54, *df* = 78, *p* < 0.05), positive emotions and life satisfaction (*r* = 0.45, *df* = 78, *p* < 0.05), and positive emotions and SWB (*r* = 0.84, *df* = 78, *p* < 0.05). There also existed a significant positive correlation between life satisfaction and spirituality (*r* = 0.39, *df* = 78, *p* < 0.05) as well as life satisfaction and SWB (*r* = 0.76, *df* = 78, *p* < 0.05). Thus, Hypothesis 2 was supported.

## Discussion

This study explored the influence of mandala drawing on SWB, mindfulness, and spirituality from positive psychology’s viewpoint. The core objectives of the research were to examine whether mandala drawing increased mindfulness, spirituality, and SWB. The results of this study provided three main conclusions. First, both CMD and IMD significantly increased the participants’ spirituality scores. However, in terms of mindfulness, no significant difference was observed in both CMD and IMD between the pre-test and post-test results. Second, CMD significantly affects the subjective well-being of subjects by significantly increasing positive affect. Third, there existed a positive correlation between spirituality, mindfulness, and SWB. Hypotheses 1a and 1b were partially supported, hypothesis 2 was supported, and these results are discussed in the following sections.

### Mandala Drawing Can Improve SWB and Spirituality

Among the two types of mandala drawing, only CMD significantly improved participants’ SWB scores, because CMD promoted the development of interpersonal relationships. Positive relationships are necessary for SWB. Loneliness has a great negative effect on life, and good relationships are the cornerstone of human happiness. To establish a good relationship is to learn to help others, and helping others is the surest way to increase happiness (Cacioppo and Patrick, 2008). For college students, peers are an important source for individuals to obtain instrumental help and emotional support, and the quality of peer relationship will significantly affect individual development and adaptation. A good interpersonal relationship can help college students to obtain a variety of effective social support; provide material, information, and spiritual support; increase their sense of belonging, pleasure, self-esteem, and self-confidence; improve interpersonal adaptability; and enhance SWB (Li et al., 2018). Good relationships help people live longer, feel happier, and have better physical and mental health. When the interpersonal relationship is harmonious, the mood will be calm and comfortable. When the interpersonal relationship is not harmonious or contradictory, they will feel nervous, anxious, and lonely. College students with a bad interpersonal relationship tend to view interpersonal conflict events from a negative perspective and exhibit bad social behavior toward others. This is not good for them to get praise from their peers and tends to lead to a variety of negative emotions, reducing subjective well-being (Rankin et al., 2018).

Between the two kinds of mandala drawing, only CMD significantly improved participants’ positive affect scores, because CMD stimulated the ventral vagus nerve, relaxed the mind, and restored the body to a state of peace and integrity. Good exercise of the social engagement in a group can activate the parasympathetic vagus nerve, enabling it to effectively regulate the level of emotional arousal and visceral state. Ultimately, it can improve a person’s ability to socialize with other people and non-verbal social communication (Liang et al., 2020). Since CMD provides a social environment that requires interpersonal and emotional interaction among its members during repeated face-to-face social interactions, it naturally promotes the development of the ventral vagus nerve. Based on this constant opportunity to exercise the neural circuits to weaken the sympathetic nervous system, thus it can inhibit the unnecessary activation of the autonomic nervous system and the ensuing fight-or-flight state (Porges, 2009). Collaborative mandala drawing can help college students to practice social skills, and the paintings can be used as a protective space for college students, because the circle of the mandala created a protection space, in which participants can use positive imagination to create and experience the calm, meet, love, happiness, and other positive emotions, and it provides the community to share and discuss them (Henderson et al., 2007).

Both types of Mandala drawing decreased participants’ negative affect scores significantly, but the effect of IMD is better. Jung believed that mandala drawing could act as a mediator between the negative emotion of a paint and the painter and between the conscious and unconscious. Mandala drawing provides an opportunity for an individual to externalize negative emotions, thereby establishing a psychological distance from the negative emotions. People realize that their negative emotions are often unmanageable and chaotic; however, mandala drawing can express these negative emotions in a healthy form (Carsley and Heath, 2020). Jung’s (2012) main purpose in using the method of mandala drawing was to establish a positive relationship between a painter and the images in his or her mind. Jung (2012) calls this approach active imagination, which involves a particular type of imaginative activity similar to dreaming with your eyes open. Another approach involves using positive emotions to distract attention from negative emotions and focus instead on the development of the positive emotions (Gruber and Oepen, 2018). Drawing divine Tibetan Buddhism symbols such as mandalas is a counterbalancing response because the mandala image painted by an individual would illustrate some characteristics of their psychological trauma. The process of painting is a process of treatment (Jung, 2012). The prerequisite for emotional regulation is the perception of emotional states, which in turn are associated with the interoception. William James was one of the first to propose a psychological theory linking visceral states to emotional experiences. He believes that emotional stimuli trigger certain visceral, vascular, or physical activity, such as changes in blood pressure and heart rate. In addition, he suggests that perception of these physical responses may be a key component in mediating emotional experiences. Although physical changes are the basis of emotional experience, the inner feelings of each person are very different (James, 2013). Feldman Barrett argued that greater sensitivity to physical states will help regulate emotional responses because ongoing physical changes can be more accurately detected, which in turn may give an advantage in distinguishing and possibly regulating different emotional states. The IMD may be a specific way of organizing self-perception of limited space, perhaps focusing on the task of organizing one’s troubled emotions into a circle. The aim is mainly to reduce the range of external perception, thus rewarding the interoception rather than the external perception (Barrett and Simmons, 2015).

Both types of Mandala drawing do not increase the participants’ mindfulness significantly. Mindfulness is an active concentration of attention that requires clear internal and external awareness. The mechanism behind this is that the individual’s attention control intensity far exceeds the controlled event, whereas in the mandala drawing process, the participant is fully immersed in the activity itself. In this state of immersion, external awareness is considerably reduced and a person’s perception of their inner self or the environment is also affected. Previous research has shown that immersion is inversely correlated with mindfulness (Sheldon et al., 2015), which may explain why mandala drawing cannot increase mindfulness significantly. Flow is a state of mind that is completely focused on a task or activity. Experiencing flow produces feelings of power, alertness, and unconsciousness. Attentional deployment is used as an emotional regulation strategy, since mandala drawing involves focusing attention on specific aspects of the situation to affect emotion (Gross, 1998). The principle of art therapy is realized through the actual manipulation of artistic materials and expresses one’s inner world in non-verbal, tactile, and visual ways (Sarid et al., 2017). Csikszentmihalyi described the state of flow in the process of art production. He found that the finished works of art are less important to the creators than to the creation process of works of art, which may be partly attributed to the inherent tactile and visual experience in artistic production (Csikszentmihalyi, 2020). Mindfulness has many similarities with avoidance mechanism, which aims at limited interaction with reality and far away from interaction with wider social conflicts. Avoidance is a negative approach in western culture, while eastern culture emphasizes the utility of this approach (Wong and Roy, 2018).

The spirituality scores of the two kinds of mandala drawing are significantly increased, but the effect of the CMD is better. In the process of mandala drawing, all kinds of creative sacred symbols have been formed, which can bring spirituality into the subconscious (Tythacott and Bellini, 2020). Spirituality is closely related to the participants’ sense of social belonging. The positive impact of mandala drawing on spirituality is due to the possibility of participants participating in the experiment that is obviously aimed at strengthening their life and belonging to the same culture (Steger, 2012). The mandala drawing may involve the situation modification of emotional regulation strategies, in which the perception of greater spirituality may influence a person’s perception, recall, or response to it. Similarly, frequent use of cognitive change, a emotion regulation strategy, can enhance the value of a particular situation and cultivate a greater sense of spirituality (Gross, 1998). Participation in spiritual activities may also increase emotional regulation, suggesting that the relationship between spirituality and emotional regulation is bidirectional. Semplonius et al. (2015) found that participation in spiritual activities provides an opportunity to regulate emotion, which in turn helps young people successfully interact with each other.

### Mindfulness, Spirituality, and SWB Are Positively Correlated

The Pearson correlation results indicated that there existed a significant positive correlation between mindfulness and SWB mainly because there existed a significant positive correlation between mindfulness and positive emotions. Positive reappraisal is considered an important mechanism for mindfulness to enhance positive emotions. Mindfulness allows an individual to respond to what is happening with a sense of disengagement from the role of the person involved. In other words, mindfulness promotes accurate assessment, allowing individuals to have space to pause and choose between multiple coping strategies. Individuals with high levels of mindfulness always respond to uncertain feelings with a positive attitude, such as seeking social support, stopping negative emotions, and solving problem centers to enhance positive emotions, thereby enhancing individual SWB (Lindsay and Creswell, 2015).

There also existed a significant positive correlation between spirituality and SWB mainly because there existed a significant positive correlation between spirituality and positive emotions. A significant positive correlation was also noted between spirituality and life satisfaction. The reason for this observation may be that when a person engages in spiritual activities, some specific areas in the individual’s brain become active. When the spiritual potential of the mind is activated, four areas of the human brain become active. These areas have evolved over several years to promote positive emotions and cognition, which in turn promote SWB (Newberg, 2014). When individuals experience spiritual ascension, the body naturally produces oxytocin, which helps people feel calm, loved, and connected. At this time, people are more willing to engage in altruistic behaviors and experience happiness (Yaden et al., 2017).

Finally, a significant positive correlation was found between mindfulness and spirituality possible because mindfulness creates a type of cognition. The individual gradually shifts from their previous focus (only on the self) to focus on a larger and more permanent part (connection with other individuals), which links the individual with a larger goal and increases their spirituality (Matiz et al., 2018).

### Research Limitations and Future Studies

This study used experimental methods to test whether mandala drawing can increase mindfulness, spirituality, and SWB. Although only some of the hypotheses were supported, our research only utilized instruments developed by western scholars. Western society is mainly influenced by Catholicism and Christian culture, whereas Chinese society is largely influenced by Confucianism, Taoism, and Buddhism. Therefore, our study of spirituality may have cross-cultural issues. It is suggested that a Chinese-based spirituality scale be developed in the future to overcome this problem. This study also only explored the influence of mandala drawing on the promotion of mindfulness, spirituality, and SWB. In the future, researchers should also examine the influence of mandala drawing on other aspects of positive psychology and work toward enhancing human happiness from all aspects.

In addition, our study method used group testing, in which the researchers paid considerable attention to the rigors of the testing procedure. However, certain participants still did not follow the instructions (e.g., answering the questions without listening to directions). The speed of completion of each participant was also different, and the participants who completed first may have interfered with the participants who had not answered all the questions. It is suggested that future research avoid group testing in this type of study to reduce the interference of participants. The participants of this study were all university students, which limits our ability to extrapolate the results to the general public. Finally, their study sample comprised considerably more female participants than male participants, which may bias the results and indicate a lack of external validity of the experiment. Therefore, it is unclear whether the results of this research are applicable to other situations and further study is warranted.

## Conclusion

This study makes several conclusions as follows. First, we found that mandala drawing not only can reduce negative emotions but also helps to raise the individual’s spiritual level, and both IMD and CMD significantly enhanced the subjects’ spirituality. Second, compared with IMD, CMD has a more significant improvement and promotion effect on SWB of subjects. In this study, it was found that CMD mainly helped to enhance SWB by affecting PA of the subjects, while IMD had no significant effect on PA, and the enhancement effect of SWB was weaker than that of CMD. Third, we have found that mindfulness, spirituality, and SWB were all have positively correlated with each other. Finally, we contribute to the mandala drawing literature by linking with mindfulness, spirituality, and SWB, and providing a more in-depth understanding of CMD.

## Data Availability Statement

The raw data supporting the conclusions of this article will be made available by the authors, without undue reservation, to any qualified researcher.

## Ethics Statement

The studies involving human participants were reviewed and approved by the Chang Gung University Ethics Committee. The patients/participants provided their written informed consent to participate in this study.

## Author Contributions

CL contributed to data acquisition, interpretation of results, and manuscript preparation. HC contributed to the experimental design, data acquisition, statistical analysis, and interpretation of results. C-YL conducted data acquisition, contributed to preparation of the experimental sites, and cooperated with data acquisition. R-TL participated in data acquisition and contributed to the interpretation of results, conceived the study, and participated in the interpretation of results. W-KC conceived and designed the study and contributed to the interpretation of results and manuscript preparation. All authors contributed to the article and approved the submitted version.

## Conflict of Interest

The authors declare that the research was conducted in the absence of any commercial or financial relationships that could be construed as a potential conflict of interest.
