# Corrigendum: Cooperative and Individual Mandala Drawing Have Different Effects on Mindfulness, Spirituality, and Subjective Well-Being

**DOI:** 10.3389/fpsyg.2020.620699

**Published:** 2020-11-19

**Authors:** Chao Liu, Hao Chen, Chia-Yi Liu, Rung-Tai Lin, Wen-Ko Chiou

**Affiliations:** ^1^College of Aviation, Hua Qiao University, Xiamen, China; ^2^Graduate Institute of Business and Management, Chang Gung University, Taoyuan, Taiwan; ^3^Department of Psychiatry, Chang Gung Memorial Hospital, Taipei, Taiwan; ^4^Graduate School of Creative Industry Design, National Taiwan University of Arts, New Taipei City, Taiwan; ^5^Department of Industrial Design, Chang Gung University, Taoyuan, Taiwan

**Keywords:** positive psychology, mandala drawings, mindfulness, spirituality, subjective well-being

In the original article, we neglected to include the funder “Ministry of Science and Technology (MOST), Taiwan, Grant MOST 109-2221-E-182-033-MY3 to Chao Liu, Hao Chen, Chia-Yi Liu, Rung-Tai Lin, and Wen-Ko Chiou”.

The authors apologize for this error and state that this does not change the scientific conclusions of the article in any way. The original article has been updated.

